# A Novel Epitope from CD22 Regulates Th1 and Th17 Cell Function in Systemic Lupus Erythematosus

**DOI:** 10.1371/journal.pone.0064572

**Published:** 2013-05-21

**Authors:** Jing Yuan, Miao Yu, Ai-Lin Cao, Xiao Chen, Li-Hua Zhang, You Song, Xiang Cheng, Zi-Hua Zhou, Min Wang, He-Ping Guo, Rong Du, Yu-Hua Liao

**Affiliations:** 1 Laboratory of Cardiovascular Immunology, Institute of Cardiology, Union Hospital, Tongji Medical College, Huazhong University of Science and Technology, Wuhan, China; 2 Department of Rheumatology, Union Hospital, Tongji Medical College, Huazhong University of Science and Technology, Wuhan, China; University of Southern California, United States of America

## Abstract

The published antibodies (Abs) against CD22 on B cells including Epratuzumab could inhibit B cell activation mainly through binding to C2-set Ig domain of CD22, but they are rarely reported to modulate the pathogenic CD4^+^ T cell function in systemic lupus erythematosus (SLE). Recently, it was proved that the extracellular amino-terminal V-set Ig domain of CD22 might mediate the interaction of B and T cells, but for now the exact effect of this domain on CD4^+^ T cell biology have not been identified. Thus, in this study, we screened out a peptide termed B2285 from this V-set Ig domain, developed the novel specific anti-B2285 Abs in rabbits, and investigated their effects in MRL/lpr mice with spontaneous SLE. The results showed that anti-B2285 Abs could ameliorate the disease severity obviously in spontaneous SLE mice with the decreased differentiations of Th1 and Th17 cells and no changes of Th2 and Treg cells. In co-cultured B cells and CD4^+^ T cells, this specific anti-CD22 Abs was observed to inhibit the anti-dsDNA Abs production, CD4^+^ T cells proliferation, the protein levels of T-bet and RORγt, and the mRNA levels of TNF-α, IFN-γ, IL-6 and IL-17 in CD4^+^ T cells. Moreover, the expression of CD45RO on CD4^+^ T cells could be also apparently diminished by this novel Abs. The data suggested that anti-B2285 Abs could slow SLE progression significantly by regulating Th1 and Th17 cells function via B-T cell interaction and the cytokine network regulation. The treatment against V-set Ig domain of CD22 would be a valuable therapeutic method for SLE and other autoimmune diseases.

## Introduction

Systemic lupus erythematosus (SLE) is a heterogeneous autoimmune disease with growing morbidity, increasing mortality, and poor life quality [1]. It is characterized by autoantibodies production, immune complex deposition, and subsequent multiple organ injury. B cells are generally thought to promote SLE development by producing pathogenic autoantibodies, and immunotherapy targeting B cells is considered as an attractive treatment for SLE, such as therapeutic antibodies (Abs) against CD20 and CD22. In contrast to anit-CD20 Abs, much attention is now focused on anit-CD22 Abs for the potential better curative effect and safety [2], [3].

CD22 is a B cell-specific membrane protein, and it modulates B cell receptor (BCR) signaling through its seven extracellular Ig-like domains [4]. Currently, there are just a few anti-CD22 Abs were developed and published because the function of those CD22 domains had not been completely clarified. In some experimental and clinical trials, Epratuzumab, a chimeric monoclonal antibody (mAb) binding to the C2-set Ig domain of CD22, was demonstrated to slow the progressions of SLE by inhibiting B cell activation and autoantibody production [5]. However, it was rarely reported that those pre-existing anti-CD22 Abs could modulate the function of CD4^+^ T cells which were also critical in the pathogenesis of SLE [6]. Therefore, the more valuable targets need to be developed in lupus.

Actually, CD22 has another role in mediating cell-cell adhesion by sialic acid ligands binding mechanism, which is triggered mostly through its extracellular amino-terminal V-set Ig domain [7]. In recent years, the scientists found that the ligands binding to this domain mediated the interaction of B and T cells, which then might provide us an effective drug target regulating CD4^+^ T cell function for the treatment of SLE [8], [9]. So in the present study, we selected the epitopes of the extracellular amino-terminal V-set Ig domain from CD22 and prepared different specific antibodies (Abs). After the identification and screening, the effects of the optimal anti-CD22 Abs on the progression of autoimmune diseases and its potential immune mechanisms in regulating CD4^+^ T cells were explored in SLE mouse models.

## Materials and Methods

### Ethics Statement

The study was carried out in accordance with the Guidelines for the Care and Use of Laboratory Animals (Science & Technology Department of Huibei Province, PR China, 2005). The protocol was approved by Animal Care and Use Committee of Hubei Province of China (Permit Number: 00017314). Animals were housed under specific pathogen-free (SPF) conditions with a 12 h day/night cycle at (22±2)°C and 60±5% humidity throughout the studies.

### CD22 peptides synthesis

The peptides corresponding to the sequence of the extracellular amino-terminal V-set Ig domains with high antigenic index, hydrophilicity and surface probability from mouse CD22 (49th-54th amino acids, 67^th^–74th amino acids, and 85th–93rd amino acids) were selected and synthesized in GL Biochem Ltd (Shanghai, China). These peptides were termed B2249 (Lys-Thr-Pro-Leu-Pro-Lys), B2267 (Glu-Phe-Asp-Lys-Ala-Thr-Lys-Lys) and B2285 (Lys-Thr-Glu-Lys-Asp-Pro- Glu-Ser-Glu) respectively. The purities of these peptides were more than 98%, which were determined by high performance liquid chromatography and mass spectrometry.

### Animals

Female New Zealand white rabbits aged 3 months and MRL/lpr mice with spontaneous SLE aged 10 weeks were purchased from the experimental animal centre of Chinese Academy of Science (Shanghai, China). All of the animals were kept in the pathogen-free mouse room in the experimental animal centre (Tongji Medical College of Huazhong University of Science and Technology).

### Antibody preparation

The synthesized B2249, B2267 and B2285 peptides were firstly conjugated to keyhole limpet haemocyanin (KLH) and then used to immunize New Zealand white rabbits respectively. After eight subcutaneous injections once every two weeks, the antibody titers were determined by enzyme-linked immunosorbent assay (ELISA) according to the method provided previously [10]. Each of these three different serum Abs was purified by protein A affinity chromatography and epitope-linked gel affinity chromatography in GL Biochem Ltd (Shanghai, China). The Abs purified from the rabbits only immunized with KLH were taken as the control. The final working concentration of each antibody was 1.0 mg/ml.

### Antibody screening and identification

The specific combinations of anti-B2249/B2267/B2285 Abs to mouse CD22 molecule on B cells were demonstrated by immunofluorescence, western blotting, and flow cytometry. After negative selection using mouse B cell isolation kit and the AutoMACS Magnetic Cell Sorter (Miltenyi Biotech, Germany), the purified B cells from the splenic cells of MRL/lpr mice were fixed with 4% paraformaldehyde at 4°C for 30 min and then incubated with the anti-B2249/B2267/B2285 Abs (1:1000) separately overnight, followed by fluorescein isothiocyanate (FITC)-conjugated anti-mouse CD19 Abs (Biolegend, California, USA) for 15 min at 4°C. After that, these B cells were washed and incubated with Phycoerythrin (PE)-conjugated anti-rabbit IgG antibody (1:200, Invitrogen, California, USA) for 2 h at room temperature and imaged using the Olympus FV500 confocal microscope.

To investigate the specificity of anti-B2285 Abs, B cells were incubated with this Abs and PE-conjugated anti-rabbit IgG antibody as above. The binding rate of anti-B2285 Abs was counted by flow cytometry, and the peptide neutralization antibodies (NB2285) produced by the co-incubation of 1 mg of B2285 and 1 mg of the anti-B2285 Abs in phosphate-buffered solution (PBS) for 8 h at 4°C were used to identify the specificity of this epitope. At the same time, the bind of anti-B2285 Abs to CD4^+^ T cells that did not express CD22 molecular were negatively selected by mouse CD4^+^ T cell isolation kit (Miltenyi Biotech, Germany) and used to exclude the non-specificity of this antibody.

The blockade effect of anti-B2285 Abs on the commercial anti-mouse CD22 binding to CD22 molecular on B cells were test by flow cytometry mentioned above. Briefly, purified B cells were fixed with 4% paraformaldehyde at 4°C for 30 min and then incubated with the anti-B2285 Abs (1:1000) overnight, followed by FITC-conjugated anti-mouse CD19 Abs (Biolegend, California, USA) and PE-conjugated anti-mouse CD22 (clone: 2D6, eBioscience, California, USA) for 15 min at 4°C.

The CD22 molecular internalization was investigated in viable B cells through pre-incubating with anti-B2285 Abs for 1 h at 37°C. After that, cells were washed in ice-cold PBS, and stained with PE-conjugated anti-mouse CD22 (clone: 2D6, eBioscience, California, USA) for 15 min at 4°C. It should be noted that the 2D6 mAbs and B2285 Abs recognize distinct epitopes on CD22. Then the cells were fixed with 4% paraformaldehyde at 4°C for 30 min and the surface expression of CD22 was analyzed by flow cytometry [5].

### Interventions and groups

After the identification and screening for the anti-B2285 Abs, all of the MRL/lpr mice were separated randomly into three groups and given different interventions at 12 weeks of age: (1) SLE group: mice were administered saline (100 µl per mouse, n = 20); (2) B2285 group: mice were injected with anti-B2285 Abs (100 µg per mouse, n = 20); (3) KLH group: mice were treated with anti-KLH Abs (100 µg per mouse, n = 20). The interventions were carried out by intraperitoneal injections once a week until the end of the experiment. Serum was separated from blood collected via retro-orbital bleeding and the urine was gathered in the early morning once every two weeks. These specimens were stored at −80°C for the following measurements. Animals of each group were killed at 50 weeks of age, and their kidneys, hearts, lungs and livers were removed freshly for the H&E (hematoxylin and eosin) examination, while the spleens were isolated immediately and aseptically for the flow cytometry and cell culture.

### ELISA

The levels of anti-dsDNA Abs and anti-nuclear Abs (ANA) in mouse sera or the supernatant of lymphocytes culture were determined by the ELISA kits (ADI, Texas, USA). The sensitivities of these two ELISA kits were optimized with serum samples diluted 1:100 and there was no cross-reactivity detected.

### Biochemical assay

Urinary protein concentrations were tested by Bradford method with the Coomassie plus (Bradford) assay kit (Thermo, Illinois, USA). The bovine serum albumin (BSA) was used as the standard and the working range is 100–1500 µg/ml.

### Histopathology

The kidneys were fixed in 10% phosphate-buffered formalin, and then were trimmed and embedded routinely in paraffin. After that, 5 µm sections were cut longitudinally and stained with H&E. The severity of glomerular impairment in H&E section was scored by two independent researchers separately in a blinded manner as 0, no significant findings; 1, minimal; 2, mild; 3, moderate; or 4, severe, as previously described by Olympus bh2 microscope [11]. For transmission electron microscopy (OPTONEM10C), kidney slices were fixed in 2.5% glutaraldehyde in 0.05 M Na cacodylate buffer and further treated according to the method provided by Blomqvist and et al [12]. The IgG deposits in the glomeruli were detected by immunofluorescence as follows: 5-um-thick frozen sections of kidney were blocked in 20% normal goat serum for 30 min and incubated with FITC-conjugated goat anti-mouse IgG (1:500; Southern Biotechnology Associates, Alabama, USA) for 1 h. Fluorescence intensity in 10 glomeruli per section was scored blindly on a scale of 0–3 (0, none; 1, weak; 2, moderate; 3, strong). In the meantime, the studies of histomorphological and pathological changes in the hearts, lungs and livers were also performed.

### Flow cytometry

Mononuclear cell suspensions were prepared from the spleens of MRL/lpr mice when they were killed. The analysis of CD4^+^ T cells subsets was carried out by FACS according to published protocols. In brief, the splenic mononuclear cells were stained extracellularly with FITC-conjugated anti-mouse CD4 Abs (eBioscience, California, USA), stimulated for 5 h with phorbol myristate acetate (PMA), ionomycin and monesin (eBioscience, California, USA), and fixed/permeabilized before intracellular staining with PE-conjugated anti–mouse IFN-γ, APC-conjugated anti-mouse IL-4, and PEcy7-conjugated anti-mouse IL-17 Abs (eBioscience, California, USA). Regulatory T cells were detected following the manufacturer's instructions by mouse regulatory T cell staining kit (eBioscience, California, USA). Isotype controls were given to regulate compensation and confirm antibody specificity. Samples were acquired and analyzed with a FACSCalibur (BD Biosciences, California, USA).

### Cell culture

The splenic B cells and CD4^+^ T cells were purified by negative selection using mouse B cells or CD4^+^ T cell isolation kit (Miltenyi Biotech, Germany) from MRL/lpr mice aged 50 weeks respectively. The purified B cells (2×10^6^ cells/ml) were co-cultured with isolated CD4^+^ T cells (2×10^6^cells/ml) for 5 d at 37°C /5% CO_2_ in a 12-well culture plate (Costar, New York, USA) in RPMI-1640 medium (Gibco, California, USA) containing 100 U/ml of penicillin, 100 µg/ml of streptomycin, 10% fetal calf serum (FCS) (Gibco, California, USA), 5 µg/ml anti-mouse CD3 (eBioscience, California, USA) and 3 µg/ml anti-mouse CD28 Abs (eBioscience, California, USA) with 10 µg/ml anti-B2285 Abs or anti-KLH Abs separately.

The supernatant of the co-culture system of B and CD4^+^ T cells was absorbed for the anti-dsDNA antibody assay by ELISA as mentioned above. The co-cultured B cells were depleted using mouse CD19 magnetic microbeads and an AutoMACS Magnetic Cell Sorter by positive selection (Miltenyi Biotech, Germany), and the CD4^+^ T cells left were prepared for protein and RNA extraction. The proliferations of the CD4^+^ T cells in the co-culture system were investigated by cell counting Kit-8 (Dojindo, Japan) according to the manufacturer's instructions. The expression of CD45RO on CD4^+^ T cells were detected by flow cytometry similar to the above procedure.

### Western blotting

For antibody screening and identification, the protocols were similar to those provide by Tuscano and et al [8]. Briefly, the total proteins of purified B cells or CD4^+^ T cells were extracted using protein extraction buffer (Pierce, Illinois, USA). Anti- B2249/B2267/B2285/KLH Abs were used as primary Abs (1:1000), and the specific bands were detected using ECL reagent (Thermo, Illinois, USA).

For the detections of transcription factor in CD4^+^ T cell differentiations, the total proteins of the co-cultured CD4^+^ T cells were extracted, and the protein concentration was determined by the BCA protein assay kit (Pierce, Illinois, USA). Samples containing 50 µg proteins were separated on a 10% SDS-PAGE for 2 h and electrotransferred onto nitrocellulose membranes for 1 h. The membranes were sequentially blocked in TBST containing 5% skim milk and then incubated with primary Abs against mouse T-bet (1:500, eBioscience, California, USA), GATA-3 (1:500, eBioscience, California, USA), RORγt (1:500, eBioscience, California, USA), FOXP3 (1:500, eBioscience, California, USA), and β-actin (1:1000, Cell Signaling Technology, Massachusetts, USA) at 4°C overnight. After washing, the membranes were further incubated with horseradish peroxidase-conjugated secondary antibody (1:3000, 37°C, 2 h). The target bands were finally washed and developed with super ECL reagent (Thermo, Illinois, USA). The results were semiquantitatively analyzed using densitometric methods.

### Isolation of mRNA and quantitative Real-Time PCR

Total RNA was extracted from purified CD4^+^ T cells by Trizol reagent (Invitrogen, California, USA). Single-stranded cDNA was produced by reverse transcription with the Reverse Transcriptase kit (Takara, Japan). SYBR ® Premix Ex Taq™ II (Takara, Japan) and the Applied 7000 (Applied Biosystem) were used for quantitative Real-Time-PCR. The primers for TNF-α, TGF-β, IFN-γ, IL-6, IL-10, IL-17 and β-actin were shown in Table 1. Samples were amplified in duplicate for 40 cycles and target gene expression was normalized to the expression of β-actin.

**Table 1:** Sequences of primers for Real-time RT-PCR.

Molecule		Sequence (5′- 3′)
TNF-α sense		TTCCAGAACTCCAGGCGGT
TNF-α anti-sense		TGGGCTACAGGCTTGTCACTC
IFN-γ sense		CTCAAGTGGCATAGATGTGGAAG
IFN-γ anti-sense		GCTGGACCTGTGGGTTGTTGA
IL-6 sense		TCTTGGGACTGATGCTGGTGA
IL-6 anti-sense		GCAAGTGCATCATCGTTGTTCA
IL-10 sense		GGACAACATACTGCTAACCGACTC
IL-10 anti-sense		ACTGGATCATTTCCGATAAGGC
IL-17 sense		ACACTGAGGCCAAGGACTTCC
IL-17 anti-sense		TCATGTGGTGGTCCAGCTTTC
TGF-β sense		GACCGCAACAACGCCATCTA
TGF-β anti-sense		CCTGTATTCCGTCTCCTTGGTTC
β-actin sense		AAGGCCAACCGTGAAAAGAT
β-actin anti-sense		GTGGTACGACCAGAGGCATAC

### Statistical analysis

Data were shown as the mean ± SEM. Statistical analysis was performed with one-way ANOVA, and ANA or survival data was analyzed using Kaplan-Meier curves (Log rank test) by SPSS11.0. P<0.05 was considered statistically significant.

## Results

### Selection for CD22 peptides in V-set Ig domains

**Figure 1:**
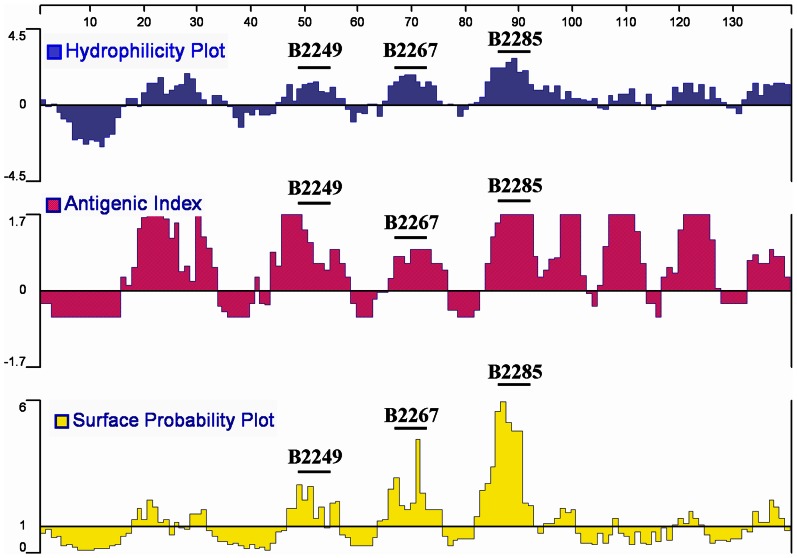
Selection for CD22 peptides in V-set Ig domains. The antigenic index, hydrophilicity and surface probability analysis were performed with DNAstar software. The sequences B2249 (49th-54th amino acids), B2267 (67th-74th amino acids), and B2285 (85th-93rd amino acids) with higher antigenic index, hydrophilicity and surface probability were selected.

Mouse CD22 contains 868 amino acids, and the continuous 109 amino acids from 26^th^ to 134^th^ are localized on V-set Ig domain (GenBank: AAD30391.1). The antigenic index, hydrophilicity and surface probability analysis were performed with DNAstar software. The sequences B2249 (49th-54th amino acids, Lys-Thr-Pro-Leu-Pro-Lys) B2267 (67th-74th amino acids, Glu-Phe-Asp-Lys-Ala-Thr-Lys-Lys), and B2285 (85th-93rd amino acids, Lys-Thr-Glu-Lys-Asp-Pro-Glu-Ser-Glu) with higher antigenic index, hydrophilicity and surface probability were selected (Fig. 1).

### Screening for anti-CD22 Abs

**Figure 2:**
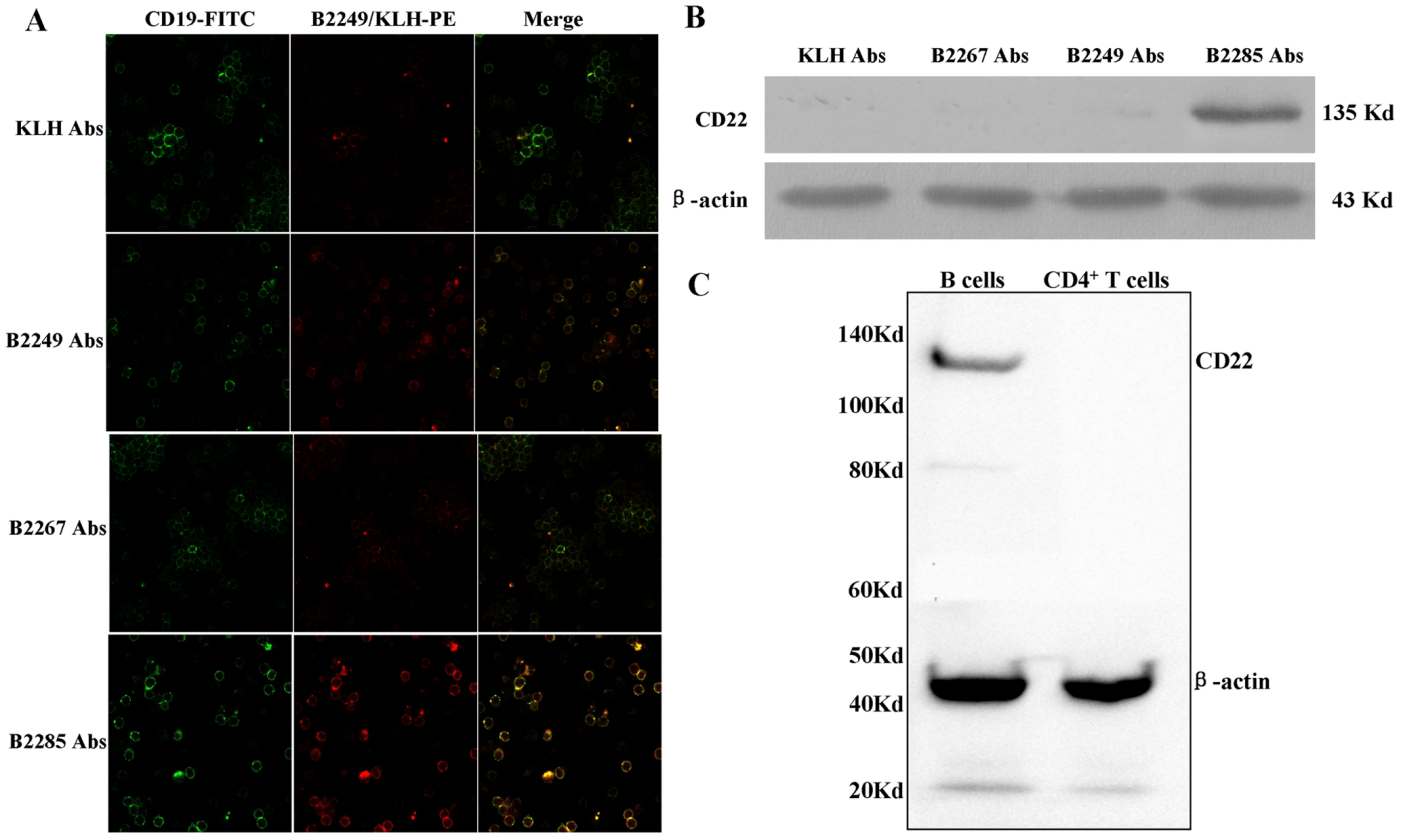
Screening for anti-CD22 Abs. (A) The immunofluorescence assay for the combination of different Abs to mouse CD22 molecular on B cells from MRL/lpr mice. The purified B cells were stained with anti-mouse CD19 Abs (green) and anti-B2249/B2267/B2285/KLH Abs (red) separately (magnification×400). The double staining of B cells (yellow) indicated the different binding affinity of four Abs to mouse CD22. (B) Western blotting analysis indicated the specific recognition of anti-B2249/B2267/B2285/KLH Abs for the CD22 protein (135 Kd) extracted from B cells. (C) Western blotting analysis indicated that anti-B2285 Abs could recognize the whole B cell lysate merely at CD22 protein site (135Kd), and did not bind to proteins from CD4^+^ T cell lysate.

We incubated purified B cells with anti-B2249/B2267/B2285 Abs respectively, and found that CD22 molecule on mouse B cells could be detected by anti-B2249 and B2285 Abs instead of anti-KLH and B2267 Abs (Fig. 2A). However, only anti-B2285 Abs could recognize and bind to CD22 protein extracted from B cells (Fig. 2B). Moreover, anti-B2285 Abs could recognize the whole B cell lysate merely at CD22 protein site (135Kd), and did not bind to proteins from CD4^+^ T cell lysate (Fig. 2C).

### Identification for the specific anti-B2285 Abs

**Figure 3:**
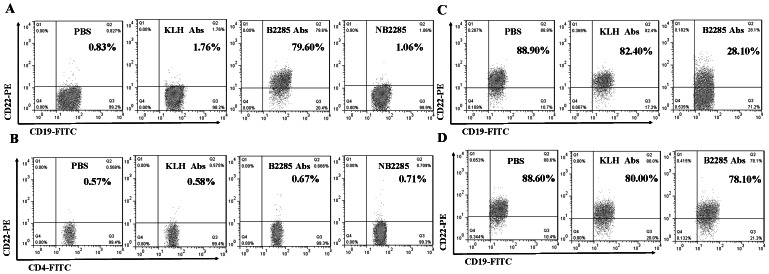
Identification for the specific anti-B2285 Abs. (A) The flow cytometry analysis for the combination of anti-B2285 Abs to mouse B cells from MRL/lpr mice in PBS, anti-KLH Abs, anti-B2285 Abs and NB2285 (B2295 peptide neutralization Abs). (B) The analysis for the combination of anti-B2285 Abs to mouse CD4^+^ T cells from MRL/lpr mice by flow cytometry. (C) The detection of CD22 internalization mediated by preincubated with different Abs on viable B cells by flow cytometry. (D) The assay for the competitive binding to fixed B cells between anti-B2285 Abs and commercial anti-mouse CD22 Abs. The percentage of CD19^+^ CD22^+^ B cells was decreased obviously in B2285 group compared with those in PBS and KLH groups.

The bind of anti-B2285 Abs to CD22 on B cells was significantly higher than those of PBS, anti-KLH Abs and NB2285 groups (P<0.01, Table 2, Fig. 3A). There were not obvious changes in the bind of anti-B2285 Abs to CD4^+^ T cells among the treatment of PBS, anti-B2285 Abs, anti-KLH Abs and NB2285 groups (Table 2, Fig. 3B). Moreover, the CD22 expression on viable splenic B cells preincubated with anti-B2285 Abs for 1 h would decreased significantly compared with those pre-incubated with PBS and anti-KLH (P<0.01, Table 2, Fig. 3C). However, for the fixed B cells that were dead and lost the ability of molecule internalization, the CD22 expression tested by the commercial anti-mouse CD22 Abs on these B cells pre-treated with PBS, anti-B2285 Abs and anti-KLH Abs showed no alterations (Table 2, Fig. 3D).

**Table 2:** Identification for the specific anti-B2285 Abs.

	PBS (%)	KLH Abs (%)	B2285 Abs (%)	NB2285 (%)
The binding rate to B cells	0.61 ± 0.09	1.45 ± 0.18	79.62 ± 6.63*	1.26 ± 0.11
The binding rate to CD4^+^ T cells	0.60 ± 0.08	0.68 ± 0.05	0.70 ± 0.07	0.73 ± 0.05
The binding rate to preincubated viable B cells	83.04 ± 2.75	79.76 ± 1.45	33.88 ± 3.38**^#^**	-
The competitive binding rate to preincubated fixed B cells	82.60 ± 2.51	79.84 ± 3.63	73.18 ± 1.97	-

* , p<0.01 vs PBS, KLH Abs and NB2285 groups; #, p<0.01 vs PBS and KLH Abs groups. Values are means ± SEM.

### The effects of anti-B2285 Abs on disease severity of MRL/lpr mice

**Figure 4:**
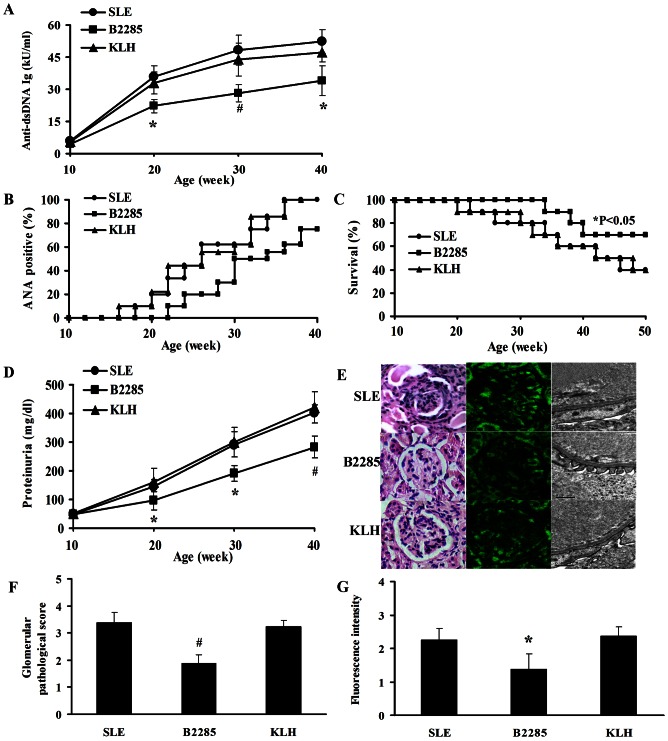
The effects of anti-B2285 Abs on disease severity of MRL/lpr mice . (A) The levels of serum anti-dsDNA autoantibodies in different groups at 10, 20, 30 and 40 weeks of age. (B) The frequencies of ANA development in different groups tested every two weeks. (C) The survival rates in different groups monitored every two weeks. (D) The levels of proteinuria in different groups at 10, 20, 30 and 40 weeks of age. (E) The representative pictures of histopathology (left, magnification×200), IgG deposition (middle, magnification×200) and transmission electron microscopy of glomerulus (right, magnification×20000). (F) The glomerular pathological scores in different groups. (G) The fluorescence intensity of IgG deposition in the basement membrane of glomeruli in different groups. *, p<0.05 vs SLE and KLH group; #, p<0.01 vs SLE and KLH group. Values are means ± SEM.

In each intervention group, the levels of anti-dsDNA autoantibodies were elevated continuously from the begin to the end of the experiment, but those in B2285 group were obviously lower than SLE and KLH groups at 20, 30 and 40 weeks of age (all P<0.05, Fig. 4A).

Serum ANA could be firstly detected in SLE and KLH groups between 16 and 18 weeks of age while it was delayed to 22 weeks in B2285 group. Furthermore, the frequency of ANA in B2285 group was reduced compared with those in SLE and KLH groups at the same time points although this improvement was not statistically significant (P = 0.08, Fig. 4B).

The death of mice in B2285 group occurred at 34 weeks of age, which was evidently later than SLE group at 22 weeks old and KLH group at 20 weeks old, and the better survival rate was also found in B2285 group compared with those in the other two groups before the end of the experiment (P<0.05, Fig. 4C).

The proteinuria, pathological score and IgG deposition in the glomeruli were carefully studied in order to evaluate the renal injury in MRL/lpr mice. In B2285 group, continuous urine tests revealed that the levels of urine protein were markedly decreased compared with those in SLE and KLH groups at 20, 30 and 40 weeks of age (all P<0.05, Fig. 4D). And at 50 weeks of age, the poliferative glomerulonephritis, podocyte foot fusion and pathological scores in B2285 group were significantly slighter or less than those in SLE and KLH groups (P<0.05, Fig. 4E and F), with the similar changes in the fluorescence intensity of IgG deposition in the basement membrane of glomeruli (P<0.05, Fig. 4E and G).

In addition, there were no differences in the levels of serum anti-dsDNA autoantibodies, ANA, the survival rate and the renal injury between SLE and KLH groups (Fig. 4A-G), and no disparities in the H&E changes of the hearts, lungs, livers between all of these three groups (data not shown).

### The role of anti-B2285 Abs in CD4^+^ T cells differentiation in MRL/lpr mice

**Figure 5:**
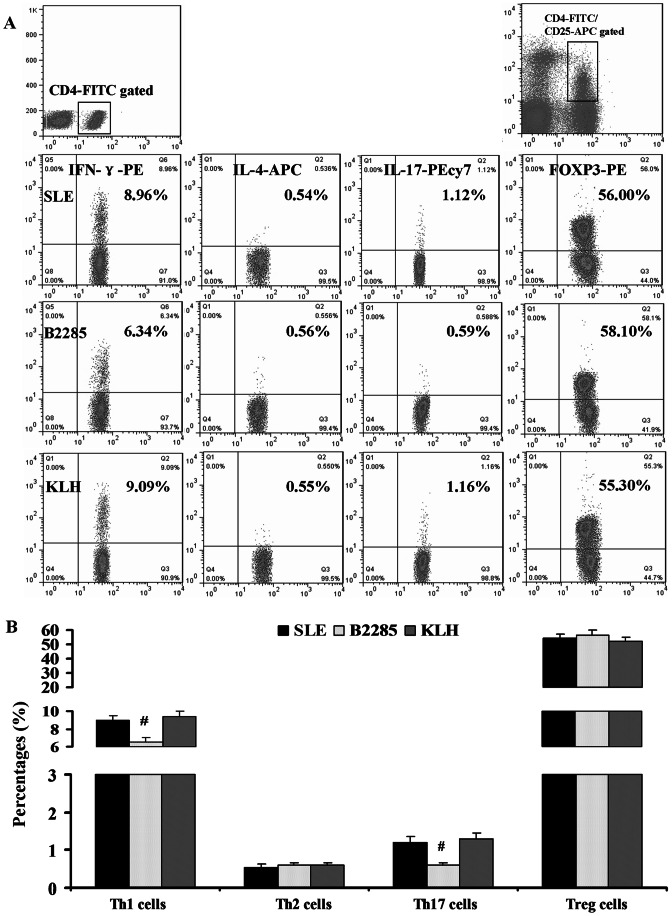
The role of anti-B2285 Abs in CD4^+^ T cells differentiation in MRL/lpr mice. (A) Representative pictures from each treatment group for Th1 (CD4^+^ IFN-γ^+^), Th2 (CD4^+^ IL-4^+^), Th17 (CD4^+^ IL-17^+^), and Treg (CD4^+^ CD25^+^ FOXP3^+^) cells. The horizontal axis exhibited the CD4^+^ cells, and the vertical axis displayed the IFN-γ^+^/IL-4^+^/FOXP3^+^ cells. Numbers in upper right quadrants indicated the percents of these cells detected respectively. (B) The results of statistical analysis for the change of Th1, Th2, Th17 and Treg cells in each group. *, p<0.05 vs SLE and KLH group; #, p<0.01 vs SLE and KLH group. Values are means ± SEM.

With the intervention of anti-B2285 Abs, the significant reductions in Th1 cells (CD4^+^ IFN-γ^+^) and Th17 cells (CD4^+^ IL-17^+^) were observed in B2285 group compared with those in SLE and KLH groups (all P<0.05). However, there were no obvious alterations in the Th2 cells (CD4^+^ IL-4^+^) and Treg cells (CD4^+^ CD25^+^ FOXP3^+^) found between these three different groups (Fig. 5A-D).

### The effects of anti-B2285 Abs on the interaction of B and CD4^+^ T cells isolated from MRL/lpr mice in vitro

**Figure 6:**
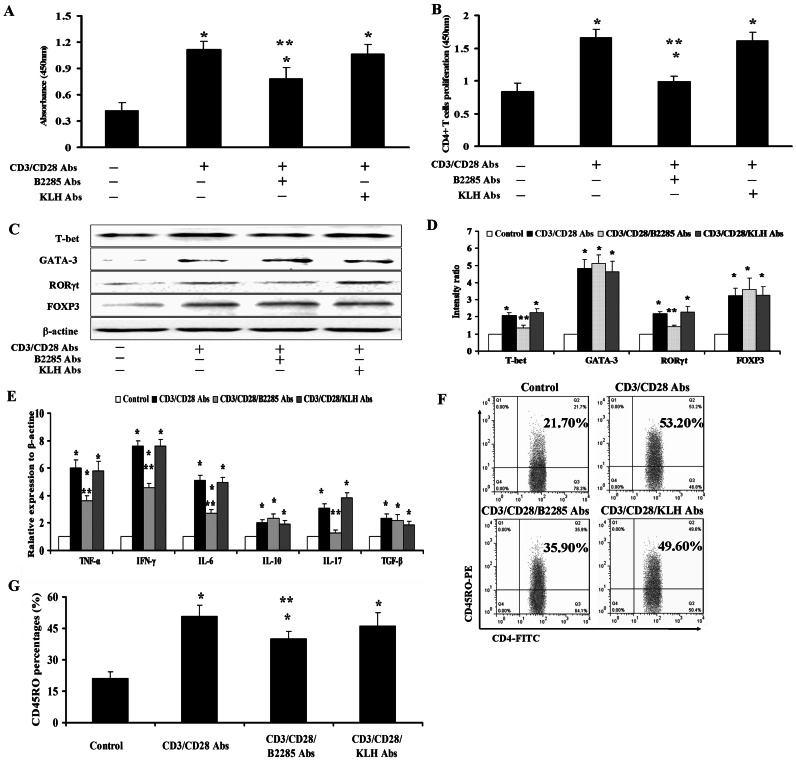
The effects of anti-B2285 Abs on the function of CD4^+^ T cells isolated from MRL/lpr mice in vitro. (A) The levels of anti-dsDNA autoantibodies in the culture supernatant from the co-cultured system of CD4^+^ T and B cells. (B) The proliferative levels of collected CD4^+^ T cells in the co-cultured system. (C) The representative pictures of T-bet, GATA-3, RORγt and FOXP3 expression in CD4^+^ T cells. (D) The results of statistical analysis for the protein levels of T-bet, GATA-3, RORγt and FOXP3 in CD4^+^ T cells. (E) The mRNA levels of TNF-α, IFN-γ, IL-6, IL-10, IL-17, and TGF-β of CD4^+^ T cells in different groups. (F) Representative pictures from each treatment group for CD45RO expression. (G) The statistical analysis for the levels of CD45RO in different groups. *, p<0.05 vs Control (medium without CD3/CD28 Abs) group; **, p<0.05 vs CD3/CD28 Abs and CD3/CD28/KLH Abs group. Values are means ± SEM.

After the CD4^+^ T cells were sorted and co-cultured with isolated B cells for 5 d, the generation of anti-dsDNA Abs and the proliferation of CD4^+^ T cells were enhanced with the stimulation of anti-CD3 and CD28 Abs than those without these stimulations. However, the levels of anti-dsDNA Abs and the proliferation of CD4^+^ T cells in this co-culture system were obviously down-regulated in the presence of anti-B2285 Abs compared with those administered with anti-KLH Abs or medium alone (P<0.05, Fig. 6A and B).

Subsequently, the co-cultured B cells were removed and the CD4^+^ T cells differentiation were investigated. The protein levels of T-bet, GATA-3, RORγt and FOXP3 in CD4^+^ T cells became higher with the stimulation of anti-CD3 and CD28 Abs than those without the participation of these two Abs (all p<0.05). And anti-B2285 Abs could remarkably diminish the elevated expression of T-bet and RORγt induced by anti-CD3 and CD28 Abs compared with anti-KLH Abs or medium alone (all p<0.05). But for GATA-3 and FOXP3 of CD4^+^ T cells, neither anti-B2285 nor anti-KLH Abs had any effect on them (Fig. 6C and D).

To further explore the immune regulative mechanism of anti-B2285 Abs, we tested the role of this Abs in the expression of the main adhesion molecule binding to CD22 and the production of some classical inflammatory cytokines in CD4^+^ T cells. Although it showed that the levels of CD45RO on CD4^+^ T cells and the mRNA of TNF-α, TGF-β, IFN-γ, IL-6, IL-10, and IL-17 in CD4^+^ T cells were all increased under the stimulation of anti-CD3 and CD28 Abs compared with those without the stimulation (all p<0.05, Fig. 6E-G), the percentages of CD45RO and the expressions of TNF-α, IFN-γ, IL-6 and IL-17 mRNA of CD4^+^ T cells could be markedly attenuated by anti-B2285 Abs while anti-KLH Abs or medium alone had no such effects (all p<0.05). Additionally, all of the increases of TGF-β and IL-10 mRNA were not changed by either anti-B2285 nor anti-KLH Abs or medium alone (Fig. 6 E-G).

## Discussion

In this study, we provided a novel therapeutic target for SLE - B2285 derived from the extracellular amino-terminal V-set Ig domain of CD22 on B cells. Antibodies against B2285 epitope could improve the disease severity in MRL/lpr mice by regulating the function of Th1 and Th17 cells.

In the course of exploring the effective drug target regulating CD4^+^ T cell function, we screened the V-set Ig domain of CD22 and designed three peptides by antigenic index, hydrophilicity, surface probability and secondary structure. It showed that only the Abs against the peptide termed B2285 could specifically bind to the CD22 molecule on B cells. Moreover, this Abs did not block the most common commercial anti-CD22 antibody from binding to CD22 on fixed B cells, but they could down-regulate CD22 expression on viable B cells after preincubate for 1 h, and the mechanism of it might be related to receptor internalization [5], [9]. Finally, this novel anti-CD22 antibody improved the disease severity, especially in the glomeruli pathological changes. Thus, B2285 was likely the novel functional epitope in the extracellular amino-terminal V-set Ig domains of CD22.

To verify the importance of B2285 in regulating abnormal immune responses of SLE and reveal the potential mechanism of anti-B2285 Abs in treating the disease, we tested and found that anti-B2285 Abs obviously inhibited the generation of anti-dsDNA Abs in SLE mice, along with the decreased Th1 and Th17 cells. Furthermore, after co-culturing of isolated B cells and sorted CD4^+^ T cells from SLE mice in vitro, anti-B2285 Abs restrained the production of anti-dsDNA autoantibodies. At the same time, the proliferation of CD4^+^ T cells and the differentiations of Th1 and Th17 cells except Th2 and Treg cells were also inhibited by anti-B2285 Abs, which were accordance with the changes of these cells in vivo. As we known, Th1, Th2 and Th17 cells were able to facilitate while Treg cells suppress autoantibodies production [13]–[15]. Although there were no changes in Th2 and Treg cells of SLE mice, the inhibition of Th1 and Th17 could impaire B cell activation, migration, maturation in the germinal centre and the production of pathogenic autoantibodies [16]–[19].

The inhibitory effect of anti-B2285 Abs on CD4^+^ T cell might be due to the modulation of B-T cells interaction or cytokine network. Firstly, we measured the expression of CD45RO that were the dominant ligands for CD22 in B-T cells interaction, and found that the CD45RO expression on CD4^+^ T cells were decreased with the reduce of CD22 expression on B cells by using anti-B2285 Abs. Then the interaction between CD22 on B cells and CD45RO on CD4^+^ T cells was declined. The decreased interactions not only attenuated the T-cell receptor (TCR)/CD3 signaling mediated by the intracellular calcium and phospholipase C1 phosphorylation, but also alleviated the T cell co-stimulatory signaling mediated by CD2 and LFA-1, which might finally lead to the impairments of CD4^+^ T cells activation, Th1/Th17 cell differentiations and the autoantibodies production [20], [21]. It indicated that B2285 played its role in Th1 and Th17 cell function probably depending on the interaction of CD22 on B cells and CD45RO on CD4^+^ T cells. In addition, anti-B2285 Abs could inhibit the mRNA expressions of TNF-α, IFN-γ, IL-6 and IL-17 in CD4^+^ T cells from the co-culture system. As these proinflammatory cytokines mostly originated from Th1 and Th17 cells, the differentiations of these two CD4^+^ T cells would be inhibited without their encouragements [22]. Nonetheless, anti-B2285 Abs could not modulate the expression of IL-10 and TGF-β mainly derived from Th2 and Treg cells, which might result in the unchanged differentiation of these two anti-inflammatory cells mediated by GATA-3 and FOXP3 [22]. These phenomena further illustrated that the effect of B2285 domain on the alterations of CD4^+^ T cell subset in SLE mice might also be attributed to the changes of cytokine microenvironments via B-T cell interaction.

In conclusion, B2285 epitope from CD22 influenced the Th1 and Th17 cells function in SLE animal models, which might result from the interaction of B-T cells and the changes of cytokine network, and the suppression of Th1 and Th17 cells contributed to slowing the progression of SLE. We thought B2285 would be a valuable therapeutic target for SLE and other autoimmune diseases, but the more studies were still needed for the accurate mechanism of anti-B2285 Abs in the immune system.
